# αO-Conotoxin GeXIVA[1,2] Suppresses In Vivo Tumor Growth of Triple-Negative Breast Cancer by Inhibiting AKT-mTOR, STAT3 and NF-κB Signaling Mediated Proliferation and Inducing Apoptosis

**DOI:** 10.3390/md22060252

**Published:** 2024-05-29

**Authors:** Xijun Guo, Leping He, Weifeng Xu, Wanrong Wang, Xiaoli Feng, Yuanfeng Fu, Xiaofan Zhang, Ren-Bo Ding, Xingzhu Qi, Jiaolin Bao, Sulan Luo

**Affiliations:** 1 Key Laboratory of Tropical Biological Resources of Ministry of Education, School of Pharmaceutical Sciences, Collaborative Innovation Center of One Health, Hainan University, Haikou 570228, China; 18273419939@163.com (X.G.); hlprin98@126.com (L.H.); 19071010110011@hainanu.edu.cn (W.X.); wangwr9@163.com (W.W.); xiaolifeng2000@163.com (X.F.); yuanfeng0414yf@163.com (Y.F.); zhangxiaofan@simm.ac.cn (X.Z.); dingrenbo@hainanu.edu.cn (R.-B.D.); qxz_hainu_edu@163.com (X.Q.); 2 State Key Laboratory of Quality Research in Chinese Medicine, Institute of Chinese Medical Sciences, University of Macau, Macao 999078, China; 3 Guangxi Key Laboratory of Special Biomedicine, School of Medicine, Guangxi University, Nanning 530004, China

**Keywords:** αO-conotoxin, GeXIVA[1,2], breast cancer, proliferation, apoptosis

## Abstract

Breast cancer is one of the leading causes of cancer mortality worldwide, and triple-negative breast cancer (TNBC) is the most problematic subtype. There is an urgent need to develop novel drug candidates for TNBC. Marine toxins are a valuable source for drug discovery. We previously identified αO-conotoxin GeXIVA[1,2] from *Conus* generalis, which is a selective antagonist of α9 nicotinic acetylcholine receptors (nAChRs). Recent studies indicated that α9 nAChR expression is positively correlated with breast cancer development; thus, α9 nAChR could serve as a therapeutic target for breast cancer. In this study, we aimed to investigate the in vivo antitumor effects of GeXIVA[1,2] on TNBC and to elucidate its underlying anticancer mechanism. Our data showed that GeXIVA[1,2] effectively suppressed 4T1 tumor growth in vivo at a very low dose of 0.1 nmol per mouse. Our results uncovered that the antitumor mechanism of GeXIVA[1,2] simultaneously induced apoptosis and blocked proliferation. Further investigations revealed that GeXIVA[1,2]-induced Caspase-3-dependent apoptosis was achieved through regulating Bax/Bcl-2 balance, and GeXIVA[1,2]-inhibited proliferation was mediated by the downregulation of the AKT-mTOR, STAT3 and NF-κB signaling pathways. Our study provides valuable arguments to demonstrate the potential of GeXIVA[1,2] as a novel marine-derived anticancer drug candidate for the treatment of TNBC.

## 1. Introduction

Cancer is an important cause of global health problems, with an estimated 19.3 million new cases and 10.0 million deaths in 2020 [1]. In total, 55.8% of all cancer deaths in the world are composed of the six leading major cancer types, including lung, colorectal, liver, stomach, breast and esophageal cancers [2]. According to statistics, the proportions of liver, stomach and esophageal burden are decreasing, while the proportions of lung, colorectal and breast cancer burden are increasing in China [3]. Among them, breast cancer is one of the leading causes of cancer mortality worldwide and the most frequently diagnosed cancer in females. Breast cancer is characterized by significant tumor heterogeneity, which hinders the selection of an appropriate treatment for individual cases [4]. Pathologists classify breast cancer into four main inherent subtypes, including luminal A, luminal B, HER2-positive and triple-negative breast cancer (TNBC), based on the expression of the following hormone receptors: estrogen (ER), progesterone (PR) and human epidermal growth factor (HER2) [5,6,7,8]. TNBC is characterized by a lack of ER, PR and HER2 expression (also defined by a lack of HER2 amplification by FISH), which accounts for approximately 15% to 20% of all breast carcinomas. Compared with other subtypes, TNBC exhibits higher metastatic potential and poorer clinical outcomes, as shown by the higher relapse and lower survival rates [9].

Nicotinic acetylcholine receptors (nAChRs) are pentameric ligand-gated ion channels, and each receptor is composed of five individual subunits. Based on the major site of their expression, nAChRs are subdivided into muscle or neuronal subtypes [10]. nAChRs are also expressed in a variety of non-neuronal cells, including cochlear hair cells [11], various immune cells and keratinocytes [12]. Previous evidence demonstrated that the activation of nAChRs could decrease the effectiveness of antitumor agents in various cancer types, such as oral cancer [13], nasal cancer [14], pancreas cancer [15], head and neck cancer [16], lung cancer [17,18,19], glioblastoma [20] and breast cancer [21]. In a case–control study, it was found that an increased risk of breast cancer was associated with the variant rs73229797 allele on the CHRNA9 gene [22]. Among breast cancer patients, there are higher expression levels of α9 nAChR in cancer tissues than in adjacent normal tissues [23]. Furthermore, a previous study showed that the overexpression of α9 nAChR was critical for promoting breast cancer metastasis [24,25]. Moreover, the knockdown of CHRNA9 blocked the growth of human breast cancer cells [24,26]. This evidence demonstrates that a9 nAChR plays an essential role in breast cancer development and would serve as a potential therapeutic target for breast cancer [27].

First identified by our group, αO-conotoxin GeXIVA, representing a new O-superfamily α-conotoxin identified from *Conus generalis* that consists of 28 amino acid residues, is a specific antagonist of α9 nAChR [28]. GeXIVA has four cysteine residues, forming three possible disulfide isomers [28]. Among them, the GeXIVA[1,2] isomer possesses the highest activity in relation to α9α10 nAChRs [29]. GeXIVA[1,2] was reported to be a potential therapeutic agent for analgesia [30,31]. Recently, our group was the first to demonstrate that GeXIVA[1,2] had anticancer activities against breast and cervical cancers in vitro [32,33,34]. Moreover, the α9 nAChR subunit was overexpressed in different breast cancer cell lines (e.g., MDA-MB-231, BT549, Bcap-37, ZR-75-30, et al.) and cervical cancer cell lines (e.g., SiHa and CaSki).

This study aims to investigate the anticancer effect of GeXIVA[1,2] in TNBC in vivo and explore its underlying molecular mechanism. We applied an allograft mouse model using the murine TNBC line 4T1 to evaluate the anticancer effects of GeXIVA[1,2] in vivo. To explore the potential anticancer mechanisms of GeXIVA[1,2], we examined several cancer-associated pathways, including apoptosis, endoplasmic reticulum stress, AKT-mTOR signaling, MAPK signaling, NF-κB signaling, STAT3 signaling and so on. Our study provides valuable arguments to demonstrate the potential of GeXIVA[1,2] as a novel marine-derived anticancer agent against TNBC.

## 2. Results

### 2.1. GeXIVA[1,2] Blocked the Growth of 4T1 TNBC In Vitro

**Figure 1:**
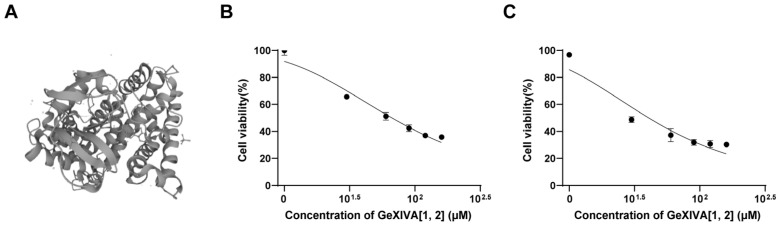
Effects of GeXIVA[1,2] on the viability of the 4T1 TNBC. (**A**) The structure of GeXIVA[1,2] (exported from UniProt database under access number J7GY56). The cells were treated with various concentrations of GeXIVA[1,2] for 48 h (**B**) and 72 h (**C**). Cell viability was determined via MTT. The values were expressed as mean ± SD of six independent assays. Statistical significance was calculated using one-way ANOVA and Tukey’s test.

GeXIVA[1,2] is an αO-conotoxin derived from marine *Conus generalis* consisting of 28 amino acid residues, with two disulfide bonds formed at Cys2–Cys9 and Cys20–Cys27 (Figure 1A) [28]. To examine its anticancer activity in relation to TNBC, we measured 4T1 mammary tumor cell growth upon GeXIVA[1,2] treatment. GeXIVA[1,2] at a dose of 30 μM remarkably decreased the viabilities of 4T1 cells to 67% and 48% after 48 and 72 h treatments, respectively (Figure 1B,C). The IC_50_ of GeXIVA[1,2] on treating 4T1 cells was 62.52 μM and 23.99 μM at 48 and 72 h, respectively. These results indicated that GeXIVA[1,2] could effectively block the growth of 4T1 TNBC in vitro.

### 2.2. GeXIVA[1,2] Inhibited the TNBC Tumor Growth in 4T1 Allograft Mice

**Figure 2:**
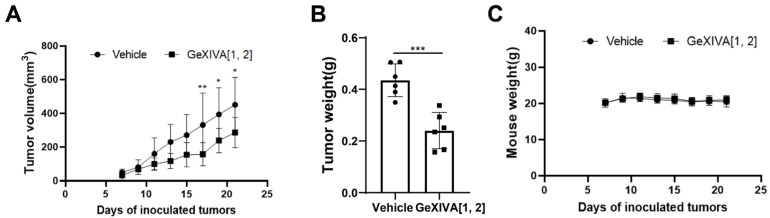
The antitumor effects of GeXIVA[1,2] on 4T1 TNBC mice. (**A**) Tumor growth curves of 4T1 allograft mice (*n* = 6) injected subcutaneously with GeXIVA[1,2] or saline. On day 0, each mouse was injected subcutaneously with 1 million 4T1 cells. Starting on day 8, GeXIVA[1,2] was injected subcutaneously once daily next to the tumor area, and tumor volume was measured every other day starting on day 7 after tumor inoculation. (**B**) Tumor weights stripped from mice on day 22 after inoculation (*n* = 6). (**C**) Mouse body weights were measured every 2 days starting on day 7. Statistical significance was calculated using one-way ANOVA, two-way ANOVA and Tukey’s test. * *p* < 0.1, ** *p* < 0.01, *** *p* < 0.001 indicates a significant difference between the treatment group and the vehicle group.

To further confirm the antitumor effect of GeXIVA[1,2] on TNBC, we treated 4T1 subcutaneous transplanted tumor mice with GeXIVA[1,2]. After administrating a 0.1 nmol dose of GeXIVA[1,2] per mouse (equivalent to 17 μg/kg) for 10 consecutive days, the tumors of mice in the GeXIVA[1,2]-treated group were significantly shrunken compared with the vehicle group, and the effective therapeutical effect of GeXIVA[1,2] lasted until the end of the experiment (Figure 2A). The obvious antitumor effect of GeXIVA[1,2] was also observed in the tumor weight results, which were significantly lower in the GeXIVA[1,2] treatment group than in the vehicle group (Figure 2B). In addition, we monitored the body weights of the treated mice and showed that the treatment of 0.1 nmol GeXIVA[1,2] did not exhibit obvious toxic effects affecting the body weights of the mice (Figure 2C).

### 2.3. GeXIVA[1,2] Induced Cancer Cell Apoptosis in 4T1 Tumors

**Figure 3:**
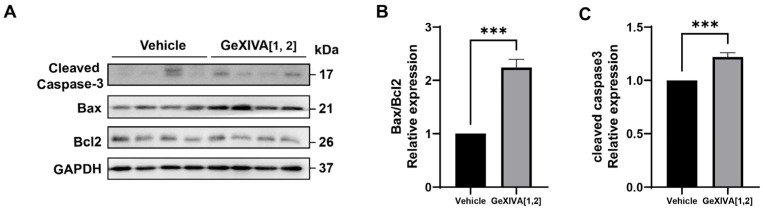
GeXIVA[1,2] induced apoptosis in 4T1 transplanted tumor model. (**A**) The expression of apoptotic pathway-related proteins (cleaved Caspase-3, Bax and Bcl2) in 4T1 tumor tissues examined via Western blot. The densitometric analysis of Bax/Bcl2 ratio (**B**) and cleaved Caspase-3 (**C**) obtained from three experimental replicates. Statistical significance was calculated using *t*-test. *** *p* < 0.001 indicates a significant difference between the treatment group and the vehicle group.

To investigate the mechanism by which GeXIVA[1,2] blocked the growth of 4T1 tumors, we first examined the expression of apoptotic pathway-related proteins in drug-treated and mock tumors via Western blot (Figure 3; Figure S1). The results showed that 0.1 nmol GeXIVA[1,2] significantly upregulated the Bax/Bcl-2 ratio and induced an obvious cleavage of Caspase-3 in the drug-treated tumors compared to the vehicle group (Figure 3). Therefore, our results indicated that 0.1 nmol GeXIVA[1,2] induced the apoptosis of cancer cells in 4T1 tumors.

### 2.4. GeXIVA[1,2]-Induced 4T1 Apoptosis Was Endoplasmic Reticulum (ER) Stress Pathway-Independent

**Figure 4:**
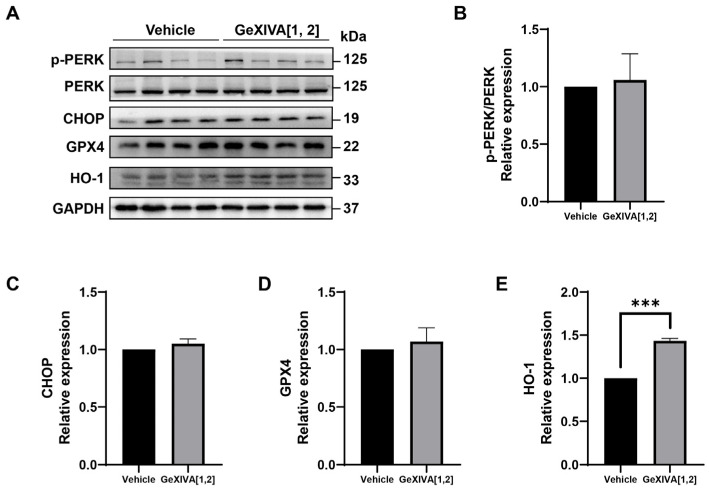
The effect of GeXIVA[1,2] on ER stress pathway-related proteins. (**A**) The expression of ER stress pathway-related proteins (PERK, CHOP, GPX4 and HO-1) in 4T1 tumor tissues examined via Western blot. The densitometric analysis of phospho-PERK/PERK ratio (**B**), CHOP (**C**), GPX4 (**D**) and HO-1 (**E**) obtained from three experimental replicates. Statistical significance was calculated using *t*-test. *** *p* < 0.001 indicates a significant difference between the treatment group and the vehicle group.

Previous studies have shown that endoplasmic reticulation (ER) stress was an important trigger of intrinsic apoptosis. Therefore, we examined the expression of ER stress-related proteins and found that all detected proteins did not show significant differences between the GeXIVA[1,2] group and the vehicle group (Figure 4A; Figure S1), including phospho-PERK/PERK (Figure 4B), CHOP (Figure 4C) and GPX4 (Figure 4D). In addition, antioxidant protein HO-1 was upregulated by GeXIVA[1,2] treatment (Figure 4E). Overall, our results suggested that GeXIVA[1,2]-induced apoptosis did not go through the ER stress pathway.

### 2.5. GeXIVA[1,2]-Induced 4T1 Growth Inhibition Was Associated with AKT-mTOR and STAT3 Signaling Pathways

**Figure 5:**
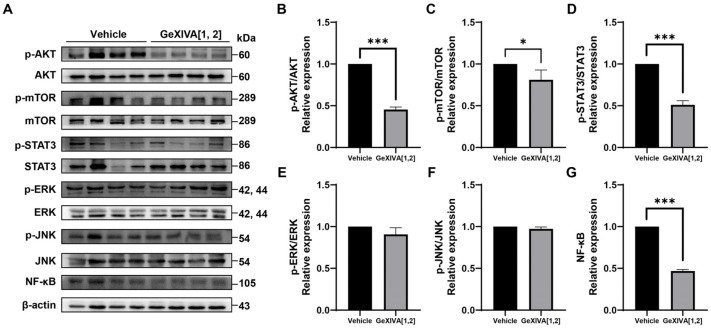
GeXIVA[1,2] inhibited proliferation in 4T1 transplanted tumor model. (**A**) Effects of GeXIVA[1,2] on the expression of proliferation upstream proteins (AKT, mTOR, STAT3, ERK, JUK and NF-κB) in 4T1 tumor tissues examined via Western blot. The densitometric analysis of phospho-AKT/AKT ratio (**B**) phospho-mTOR/mTOR ratio (**C**), phospho-STAT3/STAT3 (**D**), phospho-ERK/ERK (**E**), phospho-JNK/JUK (**F**) and NF-κB (**G**) obtained from three experimental replicates. Statistical significance was calculated using *t*-test. * *p* < 0.1, *** *p* < 0.001 indicates a significant difference between the treatment group and the vehicle group.

To further explore the mechanisms of tumor growth inhibition via GeXIVA[1,2], we examined several upstream signals for cancer cell proliferation and apoptosis (Figure 5A; Figure S1). Western blot analysis showed that phospho-AKT, phospho-mTOR, and phospho-STAT3 were downregulated by GeXIVA[1,2] treatment (Figure 5B–D), while the expressions of phospho-ERK and phospho-JNK were unchanged (Figure 5E,F). It was also found that GeXIVA[1,2] decreased the expression of NF-κB (Figure 5G). These results suggested that AKT-mTOR, STAT3 and NF-κB-mediated pathways were involved in GeXIVA[1,2]-induced 4T1 tumor growth inhibition.

## 3. Discussion

**Figure 6:**
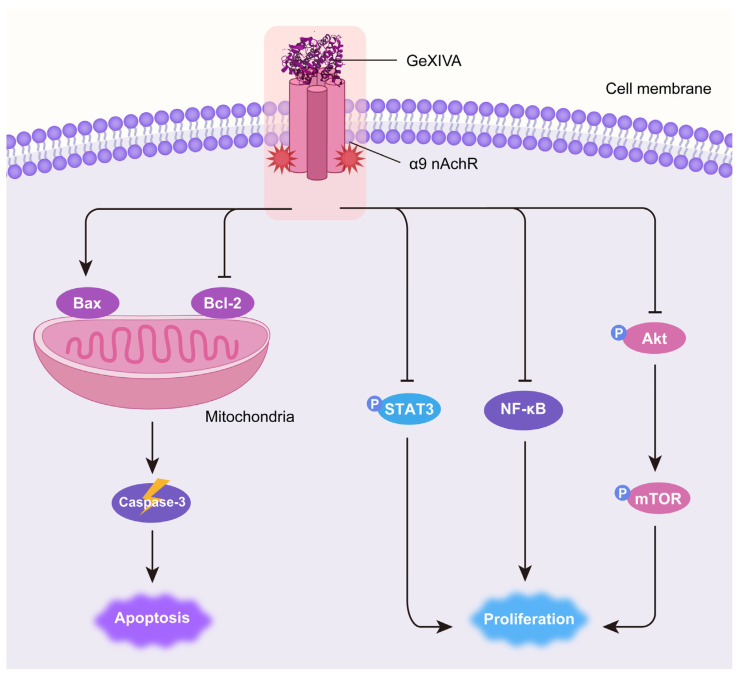
Schematic diagram of the mechanism of antitumor effects of GeXIVA[1,2] in TNBC. GeXIVA[1,2], as a specific antagonist of α9 nAChR, disrupts the balance between pro-apoptotic Bax and anti-apoptotic Bcl-2 in mitochondria, subsequently induces Caspase-3-dependent apoptosis. Meanwhile, GeXIVA[1,2] also downregulates the phosphorylation of AKT, mTOR and STAT3 as well as the expression of NF-κB, thus blocking the proliferation of 4T1 TNBC tumors.

High expression levels of α9 nAChR were demonstrated to be associated with breast cancer occurrence [24,35,36]. Sun et al. found that the expression of α9 nAChR is ~530-fold higher in MDA-MB-231 TNBC cells than that in normal mammary cells [32]. Previous studies reported that the α9 nAChR antagonist garcinol inhibited breast cancer cell proliferation [35]. α9 nAChR antagonists potentially serve as therapeutic agents for breast cancer. GeXIVA[1,2] is a specific antagonist of α9 nAChR that was first identified by our group [28]. Recently, we observed the cytotoxic effect of GeXIVA[1,2] on breast and cervical cancer cells in vitro, in which the IC_50_ for GeXIVA[1,2] on MDA-MB-157 TNBC cells was 78.31 μM [33,34]. In this study, we further demonstrated that GeXIVA[1,2] effectively suppressed 4T1 TNBC tumor growth in vivo at a very low dose. We also elucidated the antitumor mechanism of GeXIVA[1,2] by inducing Caspase-3-dependent apoptosis and inhibiting AKT-mTOR, STAT3 and NF-κB-mediated proliferation (Figure 6).

Apoptosis is a type of programmed cell death that occurs through the activation of apoptotic cascade [37]. The key players in this process are caspases, a family of cysteine-aspartic proteases involved in the initiation or execution phases of apoptosis [38]. Caspase-3 is one of the most important enzymes in the apoptotic process [39]. The activation of Caspase-3 makes apoptosis irreversible [40]. Therefore, the activation of Caspase-3 leads to specific changes in the morphology and biochemistry of apoptotic cells, which is considered a hallmark of apoptosis [41]. In our study, we found that 0.1 nmol GeXIVA[1,2] was able to induce the cleavage of Caspase-3, the activated form of Caspase-3. These results suggested that GeXIVA[1,2] induced apoptosis in TNBC cells. The classical mechanism of endogenous mitochondrial apoptosis is triggered by the mitochondrial outer membrane proteins Bax and Bcl-2 as well as the inner membrane protein, cytochrome C [42]. Upon stimulation of intrinsic apoptosis, cytochrome C is released from the mitochondria into the cytoplasm, which activates not only Caspase 3 but also the pro-apoptotic protein Bax and suppresses the anti-apoptotic protein Bcl-2 [39]. The Bcl-2 family ‘initiators’ are pro-apoptotic members, often referred to as ‘BH3-only proteins’ because most members possess only the conserved BH3 domain [43,44]. Bcl2 is an inhibitory protein of apoptosis and benefits cell survival in the absence of cytokines [45]. Bax, the guardian member of the Bcl-2 family, is often referred to as a pro-survival protein because its overexpression inhibits apoptotic cell death. In healthy cells, Bax is activated by BH3-only activator proteins (Bim and Bid). These activators are scaffolded and inhibited by anti-apoptotic Bcl-2 family members, thus preventing cell death. In the case of cells receiving overstimulation, highly expressed BH3-only sensitizers would bind to and neutralize Bcl-2 while releasing BH3-only activators to activate Bax, thereby inducing apoptosis [46,47]. An increase in the Bax/Bcl2 ratio is commonly used as a marker for apoptotic cell death. In this study, we found that 0.1 nmol GeXIVA[1,2] significantly increased the Bax/Bcl2 ratio in the tumor tissue from GeXIVA[1,2]-treated mice. Endoplasmic reticulation (ER) stress is generally considered to be an important trigger of intrinsic apoptosis. ER stress-mediated mitochondrial apoptosis was activated by endoplasmic reticulation resident proteins, cleaved ATF6 and enhanced the expression of CHOP [48]. However, there is no change in the expression of ER stress-associated proteins after GeXIVA[1,2] treatment in TNBC tumor mice. Thus, we believed the GeXIVA[1,2]-induced apoptosis was ER stress-independent.

Receptor tyrosine kinases (RTKs) are crucial upstream for cell proliferation, survival and apoptosis, in which several signaling pathways are involved, such as AKT-mTOR, JAK-STAT3, MAP kinases and NF-κB. Akt is a serine–threonine protein kinase with various downstream mediators related to proliferation, differentiation, migration, survival and metabolism [49]. The mTOR complex is comprised of two macromolecular complexes, mTOR complex 1 (mTORC1) and mTOR complex 2 (mTORC2). The activation of mTORC1 induces anabolic cell growth by promoting mRNA translation, protein synthesis, glucose metabolism and lipid synthesis. The activation of mTORC2 leads to the phosphorylation of AKT, which modulates cell survival and proliferation [50]. Akt/mTOR relays signals from several RTKs following ligand binding and regulates the release of cell proliferative promoters. Among the activation of different types of proliferative promoters, transcription factor NF-κB and STAT3 are the most prominent regulators [51,52,53]. NF-κB protein has been reported to play a key role in the development of human tumors [54]. The activation of NF-κB in cancer is believed to result in enhanced cell proliferation and invasion as well as resistance to apoptosis [55]. STAT3, a STAT family member, is an oncogenic transcription factor associated with cell survival and proliferation and regulates the expression of genes that mediate proliferation [56]. Given the role of the NF-κB and phosphorylated STAT3 (p-STAT3) in pro-survival pathways [57], we examined the effect of GeXIVA[1,2] on NF-κB and p-STAT3 and found that their expressions in tumors were downregulated by GeXIVA[1,2] treatment. Although NF-κB and p-STAT3 are regulated by different mechanisms, they appear to be simultaneously activated by Akt/mTOR. Interestingly, our results also indicated that GeXIVA[1,2] could modulate cell survival Akt/mTOR signaling cascade in 4T1 tumors. Targeting the Akt/mTOR signaling pathway can be a promising strategy for the treatment of cancer [58]. Current evidence has revealed that many polypeptide inhibitors targeting AKT, mTOR and other upstream effectors can inhibit cell proliferation [59,60,61,62]. Additionally, MAP kinases are another essential regulator for cell proliferation, differentiation and apoptosis [63,64]. Many studies have shown that the downregulation of ERK signaling could inhibit cell proliferation, and the activation of JNK signaling promotes apoptotic cell death [65,66,67]; however, we did not observe the regulatory effects of GeXIVA[1,2] on both ERK and JNK signaling in 4T1 tumor cells. Our results revealed that GeXIVA[1,2] suppressed TNBC tumor growth via the inhibition of AKT-mTOR, STAT3 and NF-κB-mediated proliferation.

Nicotinic acetylcholine receptors (nAChRs) are not only associated with the cellular regulation of tumor cell death and proliferation but have also been reported to play an important role in the regulation of immune cells [68,69]. nAChRs are expressed in various immune cells and organs, including macrophages, T cells, B cells and the spleen [70,71,72]. Subsequent studies found that the downregulation of α5 nAChR significantly increased TNF-α expression and the phagocytosis of macrophages in lung adenocarcinoma [73]. Moreover, a number of studies have reported that α7 nAChR affected the interaction between immune cells. Activated α7 nAChR was strongly associated with an increased immune cell infiltration in the tumor microenvironment and a decreased tumor/peritumor ratio of T and NK cells [74]. These previous findings indicate that nAChR is closely related to tumor immune response. Whether GeXIVA[1,2], as a specific antagonist of α9 nAChR, would play antitumor roles by affecting tumor immunity in addition to inducing apoptosis and suppressing proliferation of tumor cells is worthy of further investigation in the future.

In addition to cancer cells, α9 nAChR is also expressed in normal mammary cells and nerve cells. Thus, the potential side effects of GeXIVA[1,2] are concerning. Our previous report indicated that GeXIVA[1,2] would not induce cytotoxicity in α9-AChR expressing normal mammary cells [32]. The IC_50_ value of GeXIVA[1,2] in MCF-12A, MCF-10A and HS578BST normal mammary cells is over 200 μM, which is far higher than that in breast cancer cells [32]. These data indicated that GeXIVA[1,2]-induced cytotoxicity might be tumor-specific. Peripheral and central nerve cells highly expressed nAChRs, including α9 nAChR. GeXIVA[1,2] indeed would have an impact on α9-AChR expressing peripheral nerve cells; however, its effect is protective rather than inhibitory, which was uncovered in our previous reports [30,31,75]. GeXIVA[1,2] could protect peripheral nerve cells from chemotherapy-induced neural injury [30,31,75]. Towards the central nervous system, GeXIVA[1,2] may have a limited effect on central nerve cells because of its natural deficiency in terms of its inability to cross the blood–brain barrier via subcutaneous injection used in this study. Therefore, the potential side effects of GeXIVA[1,2] on the central nervous system are of less concern. Furthermore, we also treated the control healthy mice with GeXIVA[1,2] and found no body weight loss and other behavioral or biochemical abnormalities in these mice. In general, GeXIVA[1,2] is a relatively safe drug candidate, with no obvious side effect observed to date.

## 4. Materials and Methods

### 4.1. Cell Lines

The murine mammary carcinoma cell line 4T1 was purchased from Procell Life Science & Technology Company Limited (Wuhan, China). 4T1 cells were maintained in RPMI-1640 (Procell, PM150110) and supplemented with 10% fetal bovine serum (164210, Procell) and 1% penicillin/streptomycin (PB180120, Procell).

### 4.2. Peptide Synthesis

The synthesis and purification of GeXIVA[1,2] were carried out as previously described [28]. In short, the linear peptide with side-chain protection was synthesized using the Fmoc chemistry solid-phase methodology, and a two-step oxidation method was used to connect the disulfide bridge [76]. UPLC and LC-MS were used to confirm the molecular weight (3452.92) and purity of the compound [28].

### 4.3. Animal Model

Then, 6-week-old BABL/C female mice were purchased from Guangzhou Yancheng Biotechnology Company Limited (Guangzhou, China). The mice were maintained on a standard diet with water ad libitum at 23 °C and 30–70% relative humidity in a 12 h:12 h light–dark cycle. 4T1 cells were subcutaneously inoculated into each mouse. The tumor volumes and body weights of the mice were recorded every other day starting on day 7 after tumor inoculation. The tumor volume was calculated using the following formula: volume = (Length × Width^2^)/2. 0.1 nmol GeXIVA[1,2] per mouse (equivalent to 17 μg/kg) was administered to the mice in the GeXIVA[1,2]-treated group subcutaneously adjacent to the tumor every day starting from day 8. On the 22nd day, all mice were sacrificed, and their tumors were removed. All animal procedures were performed under the guidelines approved by the Institutional Animal Care and Use Committee of Hainan University (HNUAUCC-2021-00009).

### 4.4. In Vitro Cytotoxicity Assay

To determine the cytotoxic potential of GeXIVA[1,2], 8000 4T1 cells were seeded into 96-well plates and cultured overnight, and treated with different concentrations of GeXIVA[1,2] (0, 5, 10, 20, 40, 60, 80, 100, 120, and 160 μM) for 48 and 72 h. The supernatant was removed, and 0.5 mg/mL MTT was added at a dose of 100 μL/well. Then, the plates were incubated at 37 °C for 4 h. After that, the supernatant was discarded, and DMSO was added into the well plates at 100 μL/well. Cell viability was determined by absorbance at 570 nm using a SpectraMax^®^ M2 microplate reader (Molecular Devices, Sunnyvale, CA, USA). The mean and standard deviation of cell viability were determined and converted to the percentage of viable cells relative to the control. The dose–effect curve and IC_50_ calculation of GeXIVA[1,2] were processed by using the GraphPad Prism 9.0 software. The drug concentrations were first converted to logarithmic form, then the dose–response curves were fitted using the “nonlinear regression (cured fit)” function with the “[inhibitor] vs. Normalized response--Variable slope” mode. The formula for the fitting function is as follows: Y = Bottom + (Top − Bottom)/(1 + 10^(LogIC50 − X) × HillSlope^). X: log of dose or concentration; Y: Response, decreasing as X increases; Top and Bottom: Plateaus in same units as Y; logIC_50_: same log units as X; HillSlope: Slope factor or Hill slope, unitless.

### 4.5. Western Blot

The tissue lysates were prepared using 5× loading buffer, followed by electrophoresis on SDS-PAGE, and transferred onto polyvinylidene fluoride (PVDF) membranes (SEQ00010, Merck Millipore, Darmstadt, Germany). The membranes were incubated overnight with the following appropriate primary antibodies: cleaved Caspase-3 (1:1000, AF7022, Affinity Biosciences, Changzhou, China), Bax (1:2000, 50599-2-Ig, Proteintech, Wuhan, China), Bcl-2 (1:1000, AF6139, Affinity Biosciences), p-PERK (1:1000, DF7576, Affinity Biosciences), PERK (1:1000, A18196, ABclonal, Wuhan, China), ATF4 (1:1000, DF6008, Affinity Biosciences), CHOP (1:1000, AF6277, Affinity Biosciences), HO-1 (1:100, sc-136960, Santa Cruz Biotechnology, Dallas, TX, USA), GPX4 (1:1000, 67763-1-Ig, Proteintech), GPX4 (1:1000, 67763-1-Ig, Proteintech), p-AKT (1:1500, 66444-1-Ig, Proteintech), AKT (1:2000, 60203-2-Ig, Proteintech), p-mTOR (1:1000, AF3308, Affinity Biosciences), mTOR (1:5000, 66888-1-Ig, Proteintech), p-STAT3 (1:200, sc8059, Santa Cruz Biotechnology), STAT3 (1:200, sc8019, Santa Cruz Biotechnology), p-ERK (1:1000, AF1015, Affinity Biosciences), ERK (1:1000, BF8004, Affinity Biosciences), p-JNK (1:1000, AF3318, Affinity Biosciences), JNK (1:1000, 66210-1-Ig, Proteintech), NF-κB (1:1000, AF5006, Affinity Biosciences), GAPDH (1:10,000, 10494-1-AP, Proteintech), and β-actin (1:5000, AF7018, Affinity Biosciences) at 4 °C. Then, these were incubated with secondary antibodies such as anti-mouse (1:5000, SA00001-1, Proteintech) and anti-rabbit (1:5000, SA00001-2, Proteintech) antibodies for 1 h at room temperature. The membranes were detected using ECL (WBKLS0500, Merck Millipore).

### 4.6. Statistical Analysis

GraphPad Prism was adopted for all the statistical analysis. Pairwise comparisons were performed via one-way ANOVA, two-way ANOVA, Tukey’s test, and *t*-test. All data with *p* < 0.05 were considered statistically significant (* *p* < 0.05; ** *p* < 0.01; *** *p* < 0.001; ns, not significant (*p* > 0.05)).

## 5. Conclusions

We are the first to demonstrate the antitumor effect of GeXIVA[1,2] in vivo. Our results showed that GeXIVA[1,2] effectively suppressed 4T1 TNBC tumor growth in vivo at a very low dose of 0.1 nmol per mouse. Our data also revealed the antitumor mechanism of GeXIVA[1,2] by inducing Caspase-3-dependent apoptosis and inhibiting AKT-mTOR, STAT3 and NF-κB-mediated proliferation (Figure 6). Therefore, GeXIVA[1,2] could be a potential drug candidate for the novel treatment of breast cancer and is worthy of further study.

## Data Availability

Data will be made available upon request.

## Supplementary Materials

The following supporting information can be downloaded at: https://www.mdpi.com/article/10.3390/md22060252/s1, Figure S1: Raw data of the Western blot images.
