# Supplementary material for: αO-Conotoxin GeXIVA[1,2] Suppresses In Vivo Tumor Growth of Triple-Negative Breast Cancer by Inhibiting AKT-mTOR, STAT3 and NF-κB Signaling Mediated Proliferation and Inducing Apoptosis
Source: Mar Drugs. 2024 May 29;22(6):252. doi: 10.3390/md22060252 (PMC11205035; doi:10.3390/md22060252)

Supplementary Materials

αO-Conotoxin GeXIVA[1,2] Suppresses In Vivo Tumor Growth of Triple-Negative Breast Cancer by Inhibiting AKT-mTOR, STAT3 and NF-κB Signaling Mediated Proliferation and Inducing Apoptosis

Xijun Guo [1,†], Leping He [1,†], Weifeng Xu [1], Wanrong Wang [1], Xiaoli Feng [1], Yuanfeng Fu [1], Xiaofan Zhang [1], Ren-Bo Ding [1, 2], Xingzhu Qi [1], Jiaolin Bao [1, 2, *], Sulan Luo [1, 3, *]

[1] Key Laboratory of Tropical Biological Resources of Ministry of Education, School of Pharmaceutical Scienc-es, Collaborative Innovation Center of One Health, Hainan University, Haikou, 570228, China;
[2] State Key Laboratory of Quality Research in Chinese Medicine, Institute of Chinese Medical Sciences, University of Macau, Macao, 999078, China;
[3] Guangxi Key Laboratory of Special Biomedicine, School of Medicine, Guangxi University, Nanning, 530004, China;

* Correspondence: baojiaolin@hainanu.edu.cn (J.B.), sulan2021@gxu.edu.cn (S.L.)
† These authors contributed equally to this work

Figure S1. Raw data of the Western blot images.

Cleaved Caspase3

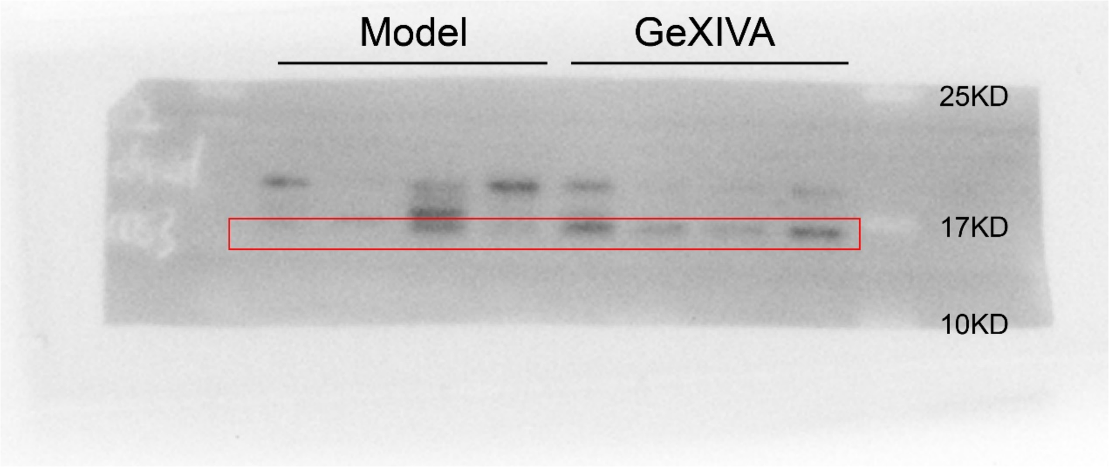

Bax

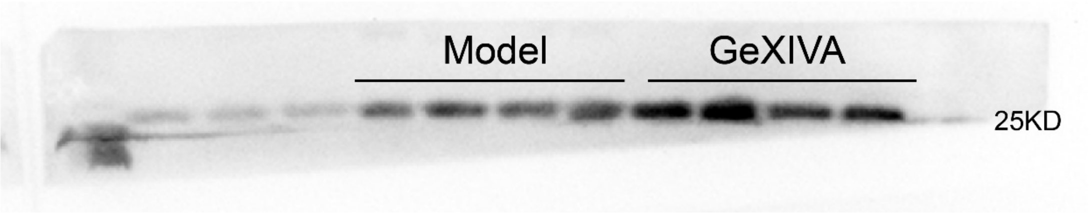

Bcl2

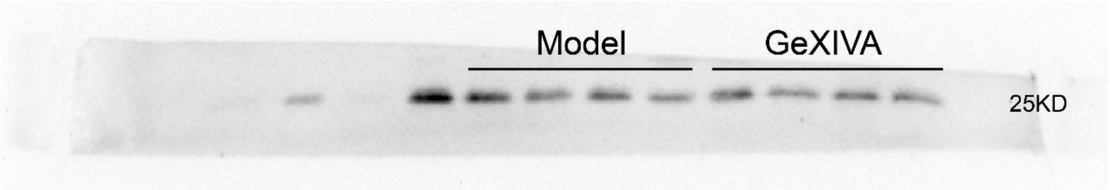

GAPDH

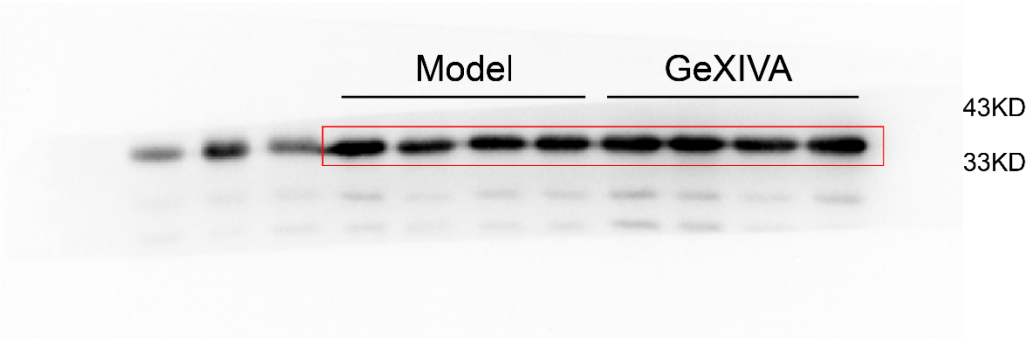

p-PERK

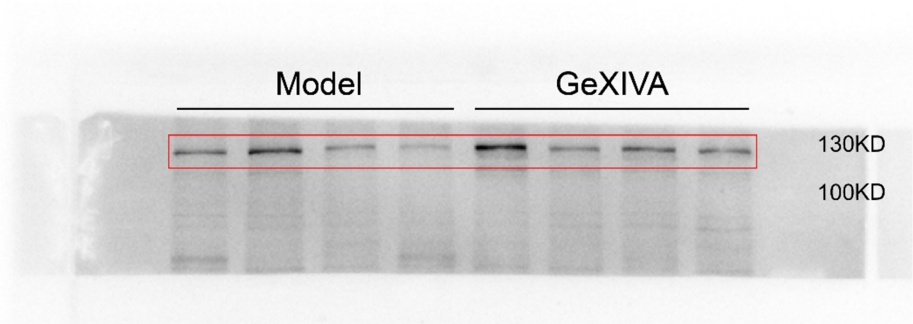

PERK

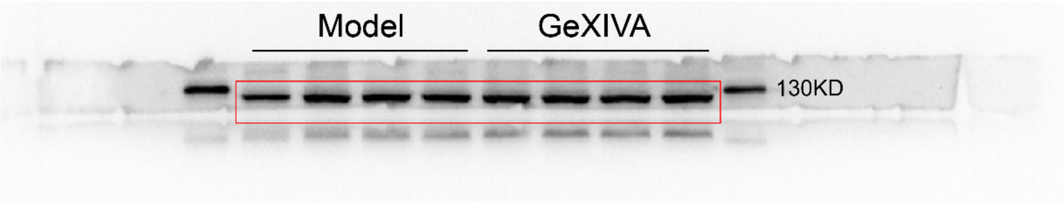

CHOP

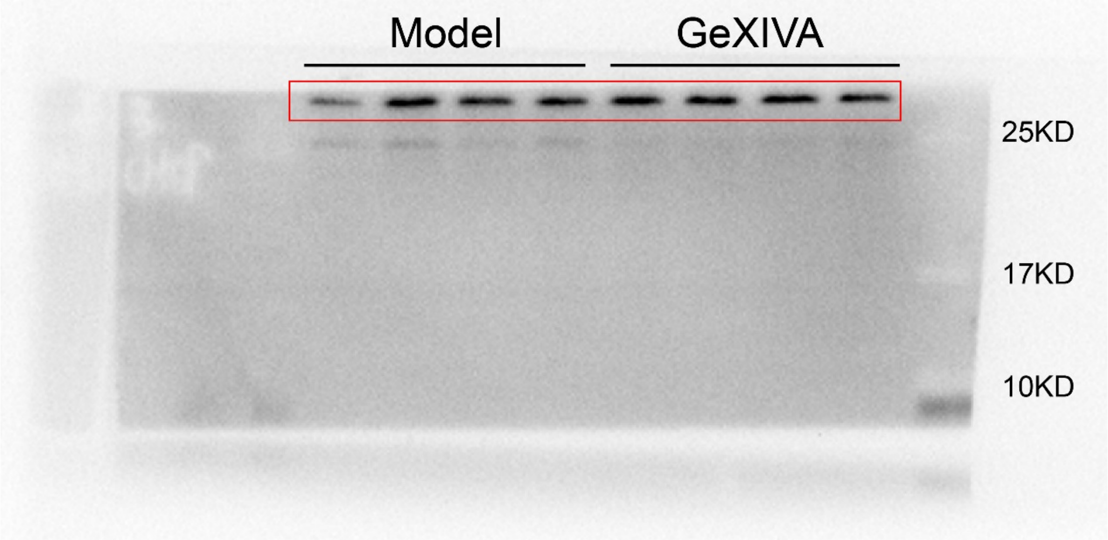

GPX4

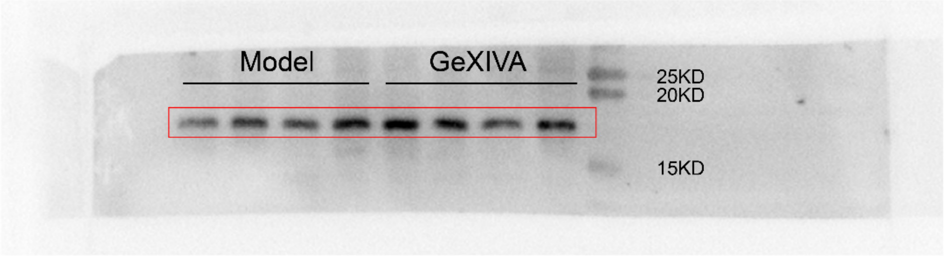

HO-1

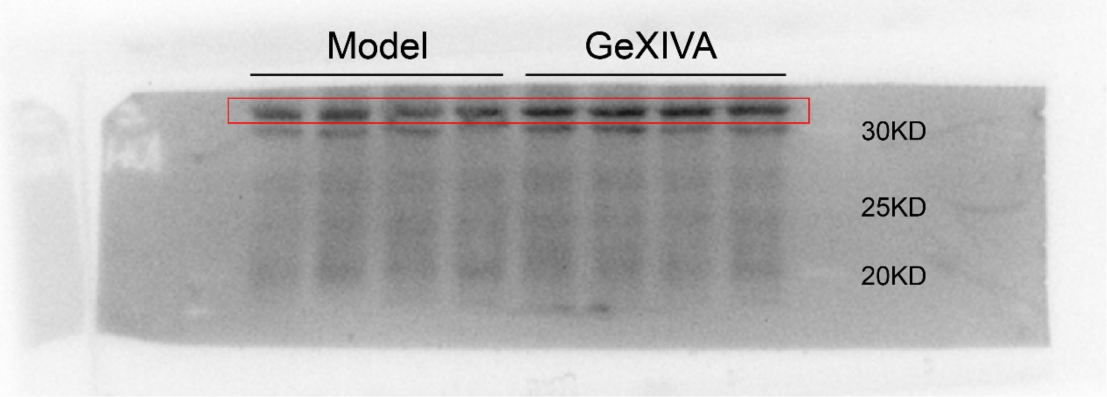

GAPDH

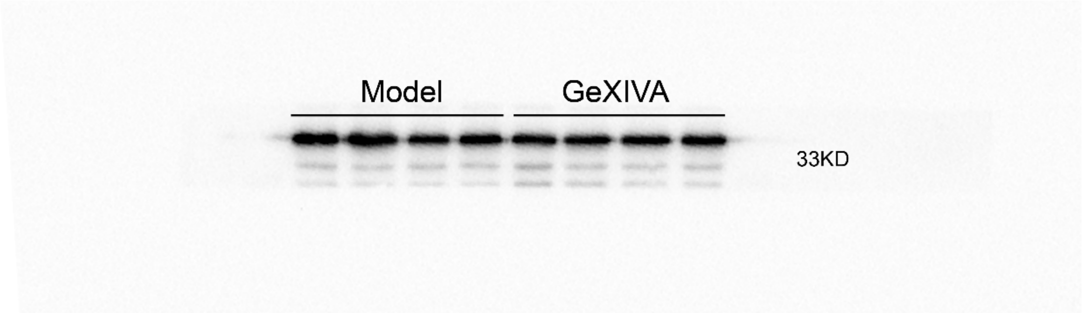

p-AKT

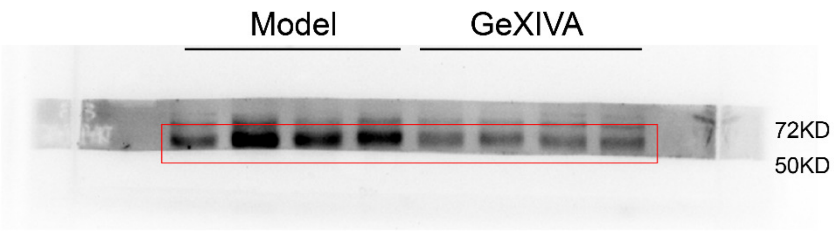

AKT

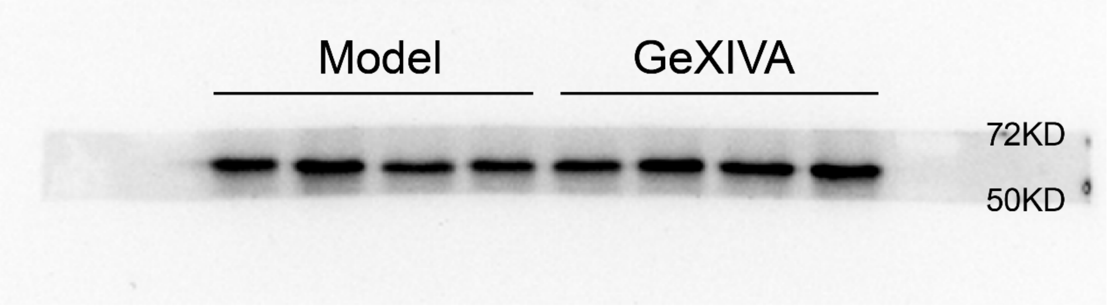

p-mTOR

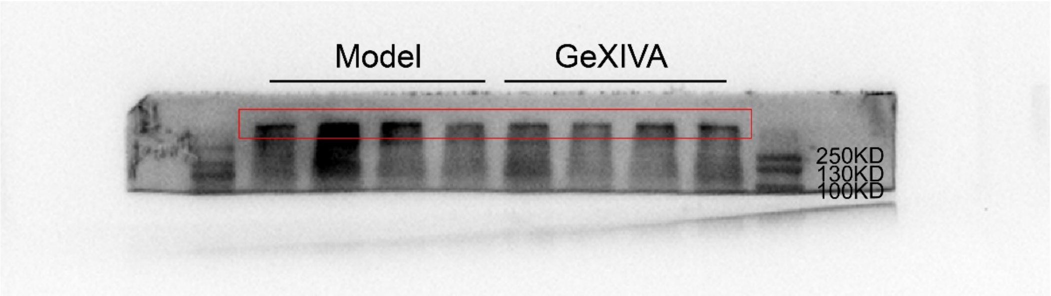

mTOR

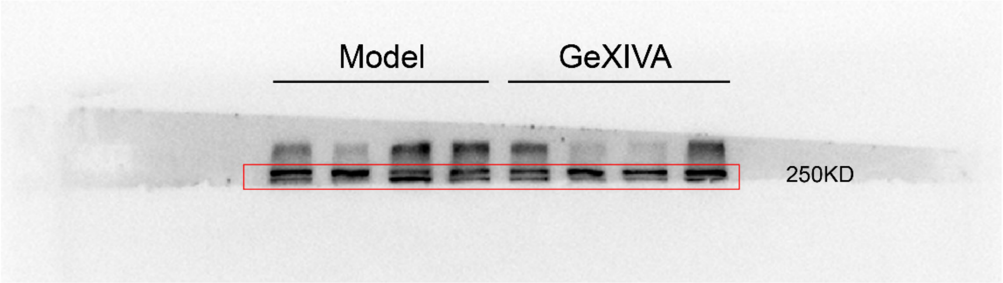

p-STAT3

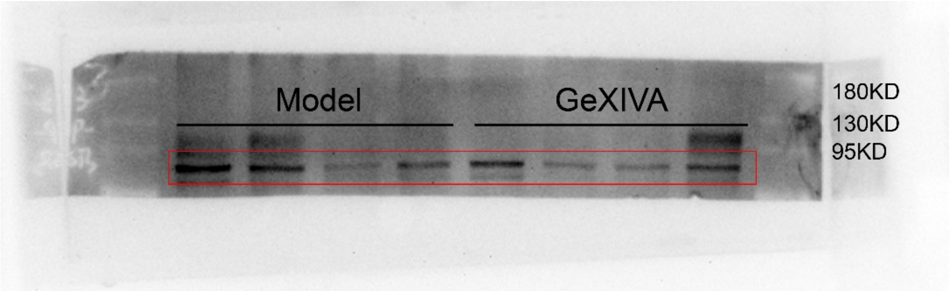

STAT3

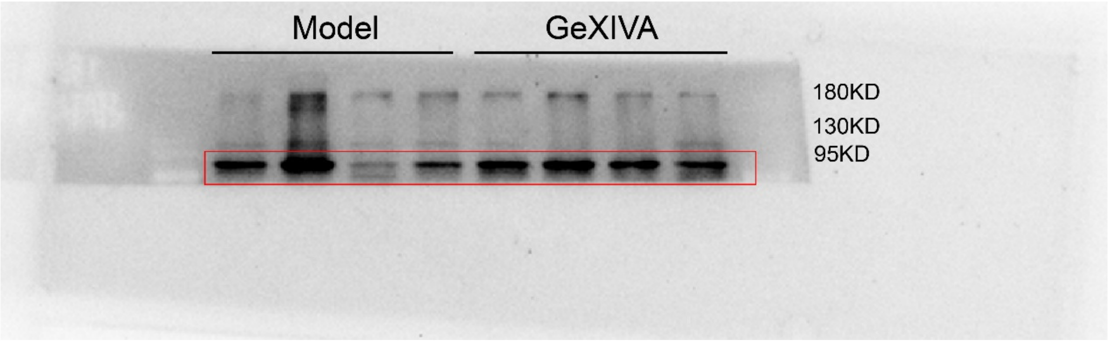

p-ERK

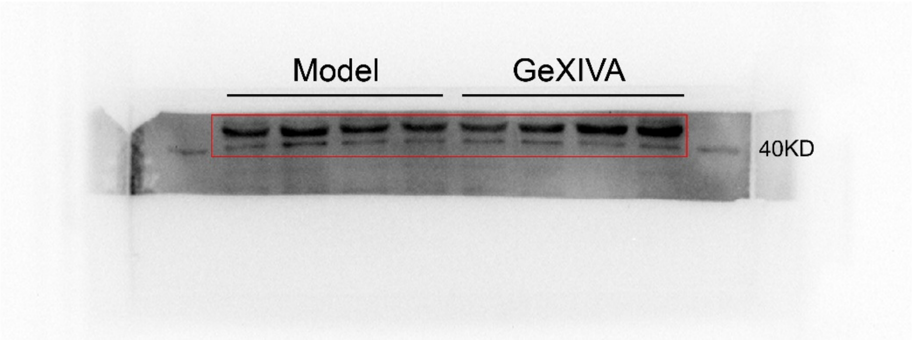

ERK

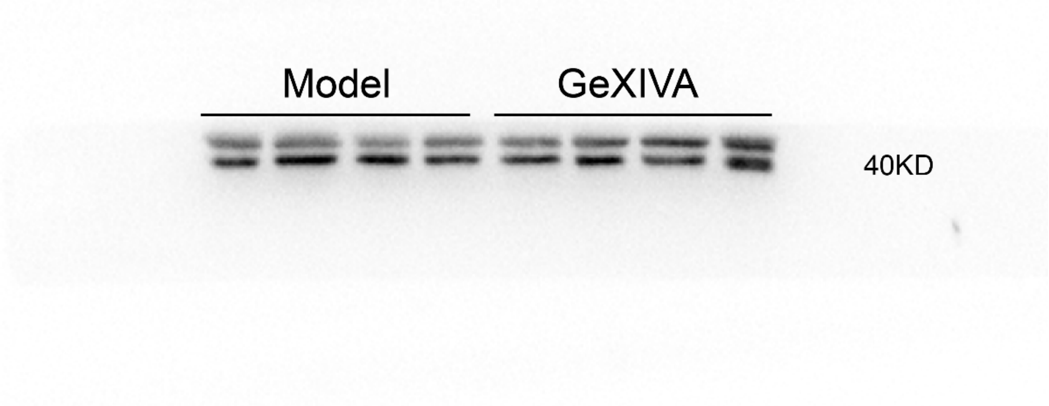

p-JNK

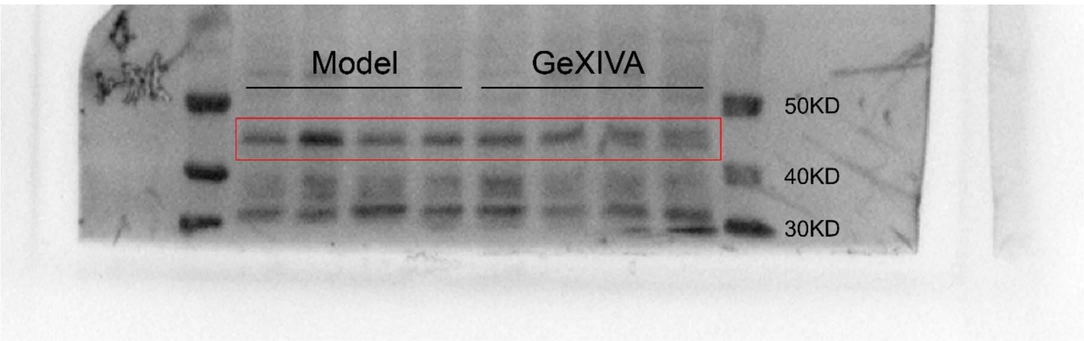

JNK

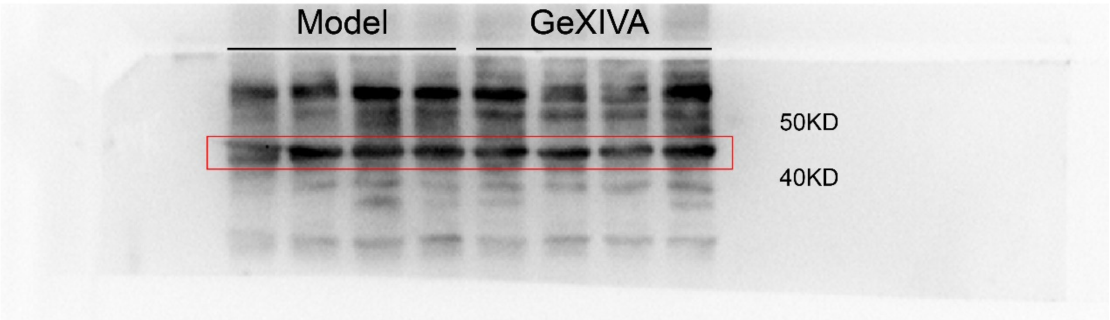

NF-κB

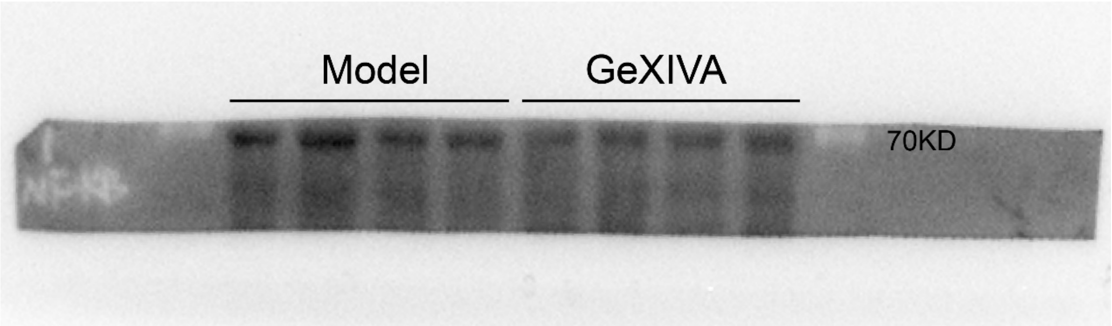

β-actin

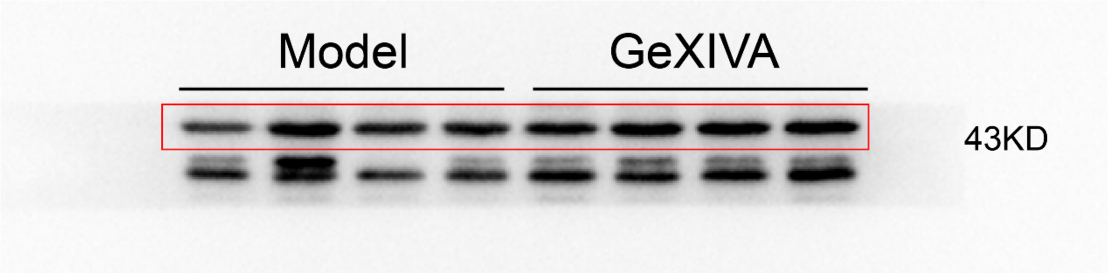

Supplement: Supplementary file 1 [file marinedrugs-22-00252-s001.zip › marinedrugs-2981237-supplementary.pdf]
